# Progress in State Medicine

**Published:** 1898-08-20

**Authors:** 


### Progress in State Medicine.

**ational Mortality.**—Much attention has recently been drawn to the subject of phosphorus poisoning by the prosecution instituted by the Home Office against Messrs. Bryant and May for concealing and failing to notify a fatal case that occurred amongst their employées. A conviction was obtained, and a penalty amounting to the sum of £25 10s. was inflicted. The case, it has been shown, was only one of a series of twelve which were not reported to the authorities. The use of yellow phosphorus in the manufacture of matches has long been recognised as a source of danger to health, and by means of free ventilation, and by removing the fumes by means of a strong draught on the off side of the dipper's plate, much has been done to minimise the risk. Still cases of phosphorus poisoning are not infrequent, and the question arises whether it is possible entirely to dispense with the use of the amorphous material, and to manufacture, with perfect safety, matches which will strike anywhere. It is claimed that the French State engineers have succeeded in giving a formula for making such matches, and the process will shortly be adopted in the Government factories. The secret has not, however, been revealed; and, in the meantime, the Government of Belgium has offered a premium of 50,000 francs to the inventor of a paste for match heads free from yellow phosphorus and capable of igniting upon any dry surface. We may hope shortly to have an authoritative statement on this matter, as Sir Matthew White Ridley has appointed Dr. Oliver and Professor Thorpe to make an expert inquiry, visiting various continental factories for the purpose. Dr. Oliver has already reported on the French works at Aubervilliers, Pantin, and Marseilles, and concludes that the reduction in the amount of illness in these places as compared with England is attributable to the greater care exercised in selecting the workpeople, to the lessened amount of child labour, better supervision, improved methods of manufacture, greater attention to personal cleanliness, and rigid medical and dental examination.

The Home Secretary reports that of 1,701 persons engaged in the dangerous processes, the cases of necrosis have been 1·07 per cent.

Since January 1st, 1896, 1,030 cases of lead poisoning have been notified.[1] Four hundred and thirty-two of these occurred from the use of lead glazes in pottery work, 239 in white lead factories, and the remainder were distributed amongst workers in glass, metals, enamels, paints, &c. Colic is the most frequent symptom, then paralysis, and then acute nervous symptoms, delirium, convulsions, and coma, which may terminate fatally in a few hours. Acute poisoning is most common in young persons, especially girls, but there are marked differences in the susceptibility of individuals. By the rules already in force no children are employed in factories for the production of white lead, nor may women be engaged in the most harmful processes. Overalls, head coverings, and respirators must be provided for the workers, exhaust fans and proper ventilators are to be used where much lead dust is produced, meals are not to be prepared or eaten in such rooms, and suitable washing accommodation is to be provided. Several new provisions have recently been introduced by the Home Office. Thus after August 1st, 1898, the minimum age of workers in dangerous parts of the industry has been raised to fourteen years, all young persons as well as women must be provided with head coverings and overalls, and shall be inspected once a month by the certifying surgeon. There shall be proper accommodation for taking meals, and at least one basin, with conveniences, with water laid on by tap, and a waste pipe, shall be provided for every five persons. It is to be hoped that the expert inquiry which is at present being held on this subject may result in the discovery of some substitute for lead in the manufacture of glazes and pigments. In the meantime the existing rules should be more rigidly enforced, and a large increase made in the number of inspectors.

Of the third of those diseases which are made notifiable by the Act of 1895—namely, anthrax—there have been several fatal cases recently reported.[2] Two rapidly fatal cases have occurred in Yorkshire, one in a man employed in emptying bales of hair and the other in a worker engaged in "drawing the fans" and sweeping out the store-rooms. In neither of these two cases had the floor been sprinkled with carbolic acid, as the order directs. It is not stated whether the men were respirators, or whether there was any skin lesion. The disease was of the pulmonary type in both instances. Since February 1st, 1898, two new regulations have been applied to the wool-sorting trade. Each workroom must have in its sorting-boards an aperture of at least four inches, with a down draught to carry off dust. Preferably, the size of the shaft should be eight or ten inches. No coat or worker's clothing may be kept as formerly in the sorting-rooms. A case of anthrax which terminated fatally is also reported from the Parcels Post Depôt, Clerkenwell. A man, aged 53, was employed in fixing hinges and fastenings on packages and baskets. Raw, untanned hide was used for the purpose, and no effort was made to render it aseptic. The worker's fingers were contaminated from the hides, and he inoculated himself by scratching a pimple on the neck. A similar case was reported from the Post Office nine years ago, and the authorities are now framing a code of special rules to prevent further accidents.

Of less frequent occurrence are the fatal accidents from gaseous poisoning, such as were recently reported from nitric acid fumes, or by the vapour of petroleum.[3] In the latter case, the workmen employed at Marseilles, in emptying a badly ventilated oil tank steamship, were attacked with tremors, extreme agitation, and excitement, with cries, stuttering speech, and wild gesticulations. These symptoms were followed by comatose sleep, with loss of memory on awakening. The subject of carbon-monoxide poisoning, which is the chief source of danger in the disastrous fires which so frequently occur in mines, has been the subject of a searching inquiry in connection with the Snaefell mining disaster. The official report[4] describes how a party of thirty men, descending the mine by ladders, were suddenly conscious of difficulty in breathing. They turned to escape, but it was found that the more hurried their efforts the greater became the breathlessness, muscular weakness, and palpitation. The younger men, whose exertions had been the greatest, were the first to be overcome, and, in all, nineteen out of thirty succumbed. The external appearances were characteristic of death by carbon-monoxide poisoning, and a sample of air from the shaft showed that rather more than 1 per cent. of carbonic oxide and more than 4 per cent. of carbonic acid were present. In subsequent explorations Dr. Haldane's mouse test was employed, and proved of great service. This test depends upon the fact that a mouse reacts to carbonic oxide much more quickly than a man, and thus timely warning is obtained. Rats were found to take twice as long to react as mice. In addition to breathlessness, pain over the heart, and muscular weakness, the symptoms are a gradually increasing indifference and disinclination for muscular effort, loss of time-sense, and subsequent unconsciousness. One point strongly emphasised by the report is that, in an atmosphere poisoned by carbonic oxide, any great exertion is calculated to bring on secondary cardiac failure; and that had the ascent been made more slowly more men would have escaped; any gain of time by increased effort being more than counterbalanced by the effects upon the heart, and by the larger absorption of gas consequent upon the deeper and more frequent respirations. The treatment recommended is perfect rest, hypodermic injections of ether, warmth, artificial respiration, and, where possible, the administration of oxygen.

[1] Brit. Med. Jour., July 23, 1898. [2] Brit. Med. Jour., May 29, 1898, &c. [3] Mabille, Soler, and Tronchet, Revue d'Hygiène. [4] Blue Book, Prof. Le Neve Foster.

